# The role of public health scientists within the German political discourse on the COVID-19 pandemic: Julia Piel

**DOI:** 10.1093/eurpub/ckac129.462

**Published:** 2022-10-25

**Authors:** M von Köppen, J Piel, C Apfelbacher

**Affiliations:** 1 ISMHSR, Otto-von-Guericke-Universität Magdeburg, Frankfurt, Germany; 2 Gesundheitswissenschaften, Hochschule Fulda, Fulda, Germany

## Abstract

**Background:**

During the COVID-19 pandemic politics was in search of scientific evidence to underpin decision making like never before. It is remarkable that voices from public health were less noticeable than those of virologists or immunologists. The aim of our ongoing study is to explore how public health scientists perceive their role in the relationship of their discipline and politics.

**Methods:**

We conducted 10 reflexive interviews with epidemiologists and public health scientists from Germany and collected documents (official statements and policy briefs of scientific societies). Data from both sources were analysed using situational analysis (Clarke, 2018), an approach used to map and analyse discourses in complex situations. To ensure data quality we used respondent validation.

**Results:**

According to participants, (1) improving population health was the top priority. Politicians tended to focus on short-term goals rather than long-term consequences. (2) Recognition of public health was increased by the pandemic in Germany. (3) However, politicians favoured virology, biomedical and clinical perspectives. (4) The strong motivation of public health scientists to support politics at the beginning of the pandemic turned into disillusionment. (5) The composition of advisory boards was described as non-transparent. (6) Initiatives by the public health community were not sufficiently impactful. (7) Expectations of policymakers regarding future cooperation were not clear to participants.

**Conclusions:**

The results present different facets of a delicate relationship between public health sciences and politics. The pandemic increased the visibility and impact of public health in Germany on the one hand but also demonstrated that the realms of public health (science) and politics were not well connected. Involving scientific expertise in politics requires more transparency and the normative assumptions underlying the logics of science and politics need to be made more explicit.

**Key messages:**

• The potential of public health to address the covid-19 pandemic has not been sufficiently acknowledged by policymakers, and the involvement of its experts requires greater transparency.

• Reflecting on the normative assumptions underlying the different logics of public health sciences and politics can support their cooperation in the future.

